# Q&A: Mesenchymal stem cells — where do they come from and is it important?

**DOI:** 10.1186/s12915-015-0212-7

**Published:** 2015-11-23

**Authors:** Iain R. Murray, Bruno Péault

**Affiliations:** BHF Centre for Vascular Regeneration, Scottish Centre for Regenerative Medicine, University of Edinburgh, Edinburgh, UK; Orthopedic Hospital Research Center and Broad Stem Cell Center, David Geffen School of Medicine, University of California, Los Angeles, CA USA

## Abstract

Mesenchymal stem — or *stromal* — cells (MSCs) have been administered in hundreds of clinical trials for multiple indications, making them some of the most commonly used selected regenerative cells. Paradoxically, MSCs have also long remained the least characterized stem cells regarding native identity and natural function, being isolated retrospectively in long-term culture. Recent years have seen progress in our understanding of the natural history of these cells, and candidate native MSCs have been identified within fetal and adult organs. Beyond basic knowledge, deciphering the biology of innate MSCs may have important positive consequences for the therapeutic use of these cells.

## What are mesenchymal stem cells, and how are they conventionally isolated?

Mesenchymal stem cells (MSCs) are multipotent stromal cells that can differentiate into a variety of lineages, including osteocytes, adipocytes and chondrocytes. This differentiation capacity, in addition to their release of trophic factors and immunomodulatory properties, holds great promise for cell therapies and tissue engineering. MSCs are not a new phenomenon. In the late nineteenth century the German biologist Cohnheim hypothesized that fibroblastic cells derived from bone marrow were involved in wound healing throughout the body [1]. In the 1970s Alexander Friedenstein, who is generally credited with the discovery of MSCs, described a population of plastic-adherent cells that emerged from long-term cultures of bone marrow and other blood-forming organs, and that he showed to have colony forming capacity and osteogenic differentiation characteristics in vitro as well as in vivo upon re-transplantation [2–4]. In light of their capacity to differentiate into bone, fat, cartilage and muscle in culture and an emerging link to the embryonic development of various mesenchymal tissues, the term “mesenchymal stem cell” was coined in 1991 by Arnold Caplan to describe these cells [5]. Cells with similar characteristics have since been found to emerge from cultures of virtually all adult and fetal organs tested [6]. Observation of these cells in culture led to a definition of MSCs by the International Society of Cell Therapy (ISCT) that included a propensity to adhere to laboratory culture plastic and the capacity to differentiate into at least bone, cartilage and fat [7]. MSCs were subsequently found to have a characteristic, although not specific, set of surface markers, with additional functions including the secretion of immunomodulatory factors and support, albeit limited, of hematopoiesis.

This body of work suggested that MSCs natively resided in all the tissues from which they were isolated; however, their exact location (whether in the stroma or, for instance, in blood vessels) was still not known. An improved understanding of the native identity and biology of these cells has recently been sought.

## Is it important to understand the native origin of MSCs?

Yes, a complete understanding of the native origin of MSCs will allow their therapeutic potential to be fully exploited. The documented multipotency, immunomodulatory and trophic effects of MSCs sparked great excitement and enthusiasm to explore the use of MSCs as progenitors in tissue engineering to replace damaged tissues of mesodermal and possibly other germ line origins, to promote regeneration, and to treat immune-mediated disease [8]. As such, the number of clinical trials using MSCs has been rising almost exponentially since 2004. However, with the “gold rush” to use MSCs in the clinical setting, the question of what MSCs naturally do in bone marrow and other tissues, and what intrinsic roles these populations may play in vivo, beyond how their functional traits might be harnessed in response to culture-related artificial cues or settings, were not understood. Cells were being used for therapeutic purposes without a true understanding of their native origin or function. An improved understanding of their location and function within tissues would not only satisfy scientific curiosity but also facilitate potential therapeutic targeting of these cells.

## Are MSCs artifacts of culture, or do identical cells natively reside in tissues, and if so, where?

The answer to that remained obscure for many years. As described above, MSCs have historically been isolated in culture, being selected from total cell suspensions based on their ability to adhere and proliferate for several weeks of primary cultivation. At difference with, for instance, hematopoietic stem cells, which were initially identified within mixed cell populations then increasingly enriched with markers and eventually purified to homogeneity from the bone marrow, MSCs remained for decades retrospectively isolated cells of unknown native identity, tissue distribution, frequency, or natural function in vivo [6]. Typically, the MSC description provided by ISCT in 2006 — that is, 40 years after Friedenstein’s original observations — still relied exclusively on markers defined in culture, giving no clue as to the innate character of these cells in vivo. With these cells having been only identified in a process requiring long-term culture and a definition based entirely on in vitro characteristics, it has been proposed by some that MSCs merely represent an artifact of culture. This is supported by a body of literature confirming that cell phenotypes are altered by exposure to culture products and adherence to stiff culture matrices. However, a number of large-scale studies of multiple human tissues have identified vascular pericytes (which ensheathe capillaries and microvessels in all tissues) by immunohistochemistry, and then purified those to homogeneity by flow cytometry [9]. Cultured pericytes, notwithstanding tissue of origin, turned out to be indistinguishable from conventional MSCs in terms of adherence, morphology, phenotype, proliferation rate, and developmental potential. Importantly, native expression of the MSC markers CD73, CD90, and CD105 by human pericytes is now well established, further supporting the hypothesis that both cell types are affiliated [9]. Altogether, these results designated microvascular pericytes as at least one class of tissue-resident MSCs, even though it was not known whether these perivascular cells in situ could also be functionally qualified as MSCs, or were only the precursors thereof.

## Are pericytes the only natural source of MSCs?

**Fig. 1.**
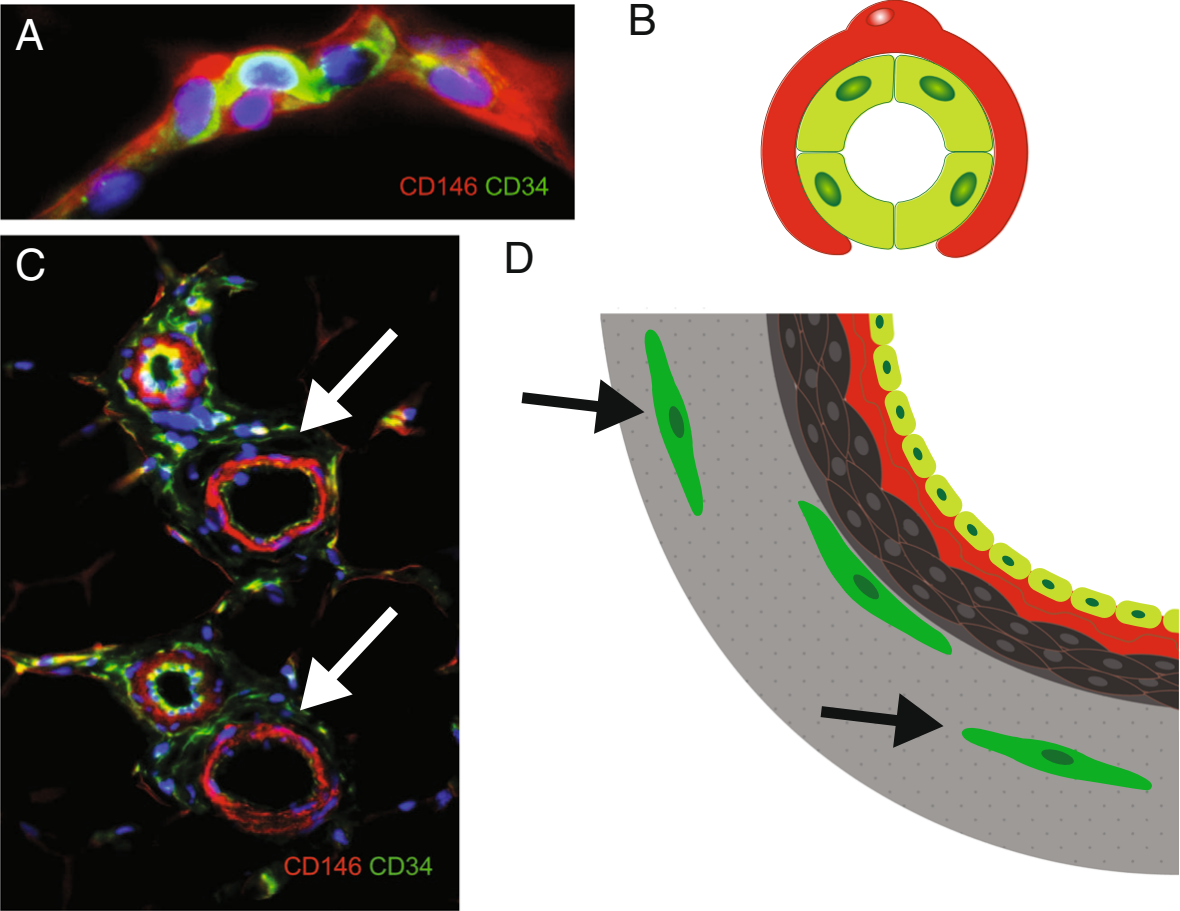
MSCs reside in perivascular locations in vivo as pericytes and adventitial cells. Fluorescent micrograph (**a**) and schematic (**b**) illustrating the intimate relationship between microvascular pericytes (CD146+CD34−) and endothelial cells (CD146+/−CD34+). Note that the *blue*/*purple color* indicates DAPI staining within nuclei of both cell types. Endothelial cells appear *yellow*/*green* because they express both CD146 (here in *red*) and CD34 (here in *green*). Adventitial cells (CD34+CD146−) located in the outmost layer of larger arteries and veins, the tunica adventitia, have also been identified as a source of bona fide MSCs (**c**, **d**). Smooth muscle cells around some larger vessels, which are perivascular but not sensu stricto pericytes, also express CD146, illustrating that marker expression must be assessed in combination with microanatomy

No. Pericytes are not the only perivascular cells endowed with potential to give rise to MSCs, which are therefore not necessarily associated with capillaries and microvessels. A population of progenitors located in the outmost layer of larger arteries and veins, the tunica adventitia, has also been identified as a source of bona fide MSCs [10]. Adventitial progenitors are phenotypically and anatomically distinct from pericytes. However, like pericytes, adventitial cells natively express MSC markers. In this regard, pericytes and adventitial cells have been collectively termed perivascular stem cells or PSCs (Fig. 1).

Although experimental evidence points to a perivascular origin of culture-selected MSCs, the possibility that non-vascular cells in some tissues can yield MSCs in culture cannot be excluded. In support of this it has recently been observed that a subset of osteogenic progenitors that exhibit MSC potential reside within cranial sutures but do not appear to be associated with blood vessels [11]. However, it remains to be demonstrated that such progenitor cells are not affiliated with perivascular cells that emerged earlier in development, but eventually migrated away from the vascular niche.

## How do native MSCs support tissue regeneration?

Although the multipotent, immunomodulatory and trophic effects of MSCs demonstrated in culture hold great promise for cell therapy [12], whether these MSCs also play a natural role in tissue regeneration in vivo is not yet fully established. The discovery that pericytes represent innate MSCs has greatly facilitated our understanding of the native roles of MSCs, as these cells can now be studied in vivo and their fates explored through lineage tracing using established pericyte markers [13]. Recent publications have already documented the direct involvement of perivascular progenitor cells in the development and/or regeneration of white adipocytes [14], skeletal muscle [15], follicular dendritic cells [16] and dental pulp [13]. Altogether, these observations suggest the presence in the adult of regenerative cells associated with all blood vessels, hence broadly disseminated throughout the organism. Intriguingly, pericyte contribution to musculoskeletal tissue development and regeneration is not absolute and varies with anatomical location. For example, the contribution of pericytes to myofibers varies among different muscles, ranging from <1 % (tibialis anterior muscle) to 7 % (diaphragm) of the fibers and is enhanced (although only modestly) by acute or chronic muscle regeneration [15].

The number of molecules known to mediate the paracrine action of MSCs continues to increase [17]. MSC paracrine effects demonstrated in vitro can be divided into trophic, immunomodulatory and chemoattractant [18]. The trophic effects of MSCs include the inhibition of apoptosis, support of regeneration and stimulation, maintenance, proliferation and differentiation of tissue-specific progenitors [19]. Perivascular cells have been shown to support the growth and differentiation of local stem cells in a number of settings, including ischemic brain injury [20]. Hypoxia takes place in the initial stages of tissue injury and secretion of anti-apoptotic factors by MSCs minimizes the extent of cell death in the tissues surrounding the injured areas. Re-establishment of blood supply is fundamental for recovery of damaged tissues and the pro-angiogenic effect of MSCs has been demonstrated in murine models of hind-limb ischemia [21]. Furthermore, the transition back to a pericyte phenotype serves to stabilize the forming vasculature both in vitro and in vivo [22]. The immunomodulatory properties of bone marrow-derived MSCs, including their immunosuppressive effects during allogeneic stem cell transplantation, have been well documented [23–27]. The immunoactivity of the cells is mediated by direct cell–cell contact and through secreted bioactive molecules involving dendritic cells and B and T cells, including T regulatory cells and T helper cells as well as killer cells. MSCs also secrete a variety of chemoattractant molecules. Target cells for these include monocytes, eosinophils, neutrophils, basophils, memory and naïve T cells, B cells, natural killer cells, dendritic cells and endothelial cell progenitors. It is likely that the pattern of chemokine expression by MSCs is modified by exposure to other cell types, particularly immune cells. As can be seen from above, native MSCs use a variety of processes and molecules to support tissue regeneration.

## Do native MSCs exert exclusively beneficial effects?

No. There is increasing evidence to support a central role for pericytes in tissue fibrosis [28, 29]. Fibrosis is the pathological persistent accumulation of collagenous extracellular matrix that can impair tissue function in a large variety of vital organs and tissues. Despite the diverse range of tissues susceptible to fibrosis, all fibrotic reactions share common cellular and molecular mechanisms [30]. Often starting as a beneficial physiological repair response to organ injury with hemostatic, inflammatory and remodeling phases, fibrosis is characterized by the persistent activity of matrix remodeling myofibroblasts. Several recent studies using cutting edge murine genetic cell labeling techniques have identified pericytes as major myofibroblast progenitors in multiple organs [28, 29]. Similarly, studies have emerged implicating pathological differentiation of MSCs in the development of heterotopic ossification [31]. As such it is not likely that the detrimental effects are a ‘normal’ function, rather a pathology that occurs when normal function becomes dysregulated.

## Are MSCs true stem cells?

This is a source of much dispute within the MSC literature [32, 33]. Stem cells are strictly defined by their ability to reconstruct the tissue of origin in vivo while also contributing to its maintenance and repair — thus demonstrating multipotency and an ability to self-renew. Stem cells differ from progenitor cells (or transient amplifying cells) that exhibit extended proliferative capacity and differentiation in vitro but have little ability to contribute to long-term tissue regeneration in vivo*.* Hematopoietic stem cells (HSCs) are the best characterized adult stem cells, and therefore are often used as a benchmark as to the level of evidence required to demonstrate the defining criteria of stem cells. HSCs can give rise to all blood cell components, including neutrophils, lymphocytes, natural killer cells, dendritic cells, macrophages and monocytes. MSCs derived from multiple sources have similarly been shown to give rise to osteoblasts, chondrocytes, adipocytes and reticular stroma. Stem cells also possess the ability to undergo numerous cell divisions while retaining their stem cell identity; that is, they have the capacity for self-renewal. For example, HSCs transplanted into irradiated mice can reconstitute the entire hematopoietic system while also giving rise to further HSCs that can be serially re-transplanted [34]. Transplantation of bone marrow-resident MSCs gives rise to ‘ossicles’ composed of bone, cartilage and reticular stroma, as well as to a population of serially re-transplantable MSCs [35, 36]. Despite this, there is very little evidence for long-term skeletal regeneration of MSCs in vivo. Transplantation of MSCs through intravenous infusion has resulted in little or no engraftment of cells to bone or bone marrow. However, intra-femoral injection of a subset of murine marrow stromal cells has shown limited engraftment at the site of injection 4–6 weeks post-transplantation, suggesting that the quality of the cells injected as well as the route of injection are major determinants of engraftment [37, 38]. In other words, MSCs obviously self-renew in culture, which guarantees the sustained presence of regenerative cells over many passages and, accordingly, express factors known to regulate the self-renewal of other, better characterized stem cells [39]. By contrast, whether MSCs re-transplanted in vivo behave like bona fide stem cells in terms of renewal is dubious. Regarding MSC natural origin in situ, quiescent perivascular cells presumably lose contact with blood vessels to be reprogrammed into stem/regenerative cells. Whether MSCs that arise this way also undergo self-renewal or are fully consumed in the tissue repair response is unknown. *In fine*, the self-renewing MSC might well be confined to the culture flask.

## Can native MSCs be purified without a requirement for cell culture?

Traditional methods of MSC isolation have depended on a period of cell culture for two reasons. Firstly, as the exact location or phenotype of MSCs in tissues was not known, it was not possible to target specifically these cells for purification. Instead researchers had to wait for these cells to emerge from cell culture where they would overgrow other cell types. Recent insights into the perivascular origin of MSCs combined with advances in multicolor flow cytometry have overcome this, enabling prospective purification of innate MSCs to homogeneity on the basis of established pericyte and adventitial cell markers [40]. All vascularized tissues are now possible immediate sources of purified MSCs, which can now be isolated using fluorescence activated cell sorting (FACS).

The second reason for historic dependence on culture has been the need to expand cell populations to clinically relevant numbers [12]. Low stem cell yield and donor site morbidity limit the use of fresh autologous bone marrow, periosteum and the majority of other MSC sources [6]. FACS purification of perivascular cells has highlighted the varying abundance of MSCs within different organs. Relative to the lower yield, limited donor sites, and morbidity associated with bone marrow or periosteal harvest, adipose tissue is now a well-documented, easily accessible, abundant and dispensable source of perivascular MSCs [41]. Approximately 15 million perivascular MSCs can be purified per 100 ml of lipoaspirate — a sufficient number of cells to treat a broad range of musculoskeletal disorders without culture expansion [42]. So the answer is yes, MSCs can now be isolated without the need for cell culture but not in high enough numbers to treat all current clinical applications, for example, graft versus host disease where multiple, large repeated doses are often required.

## Why bother to purify MSCs anyway?

Whole bone marrow cell suspensions and the stromal vascular fraction (SVF) of adipose tissue have been used directly in therapeutic applications with the aim of harnessing the potential of the contained stem cells. This type of stem cell therapy is heavily studied by many biotech companies developing benchtop systems that produce SVF at the bedside, which makes the cell mixture conveniently available shortly after harvesting adipose tissue through liposuction. However, both represent highly heterogeneous cell populations, which include non-MSC types, such as inflammatory cells, hematopoietic cells, endothelial cells, and non-viable cells among others. Available studies using SVF show poor and unreliable tissue formation [43], or lower tissue regeneration efficacy relative to cultured MSCs [44]. In fact, recent studies have suggested that the presence of endothelial cells has inhibiting effects on bone differentiation, among other lineages [45]. Despite the process of enrichment through plastic adherence, it is inevitable that even cultured preparations will be contaminated by non-MSC populations, and the contribution of each contained population to the repair process cannot be definitively established.

In addition, variability in cell composition presents clear barriers to approval from regulatory bodies such as the Food and Drug Administration (FDA) of future stem cell-based therapeutics, potentially including reduced safety, purity, identity, potency and efficacy [46]. With these regulatory hurdles in mind, the use of purified presumptive MSCs, that is, pericytes and adventitial cells, has clear practical advantages [12].

## Why might it be advantageous to avoid cell culture?

The selection and preparation of MSCs through adherence to culture plastic is time consuming, and introduces additional risks such as immunogenicity and infection through exposure to animal-derived culture products. Investigators have documented the influence of MSC culture on genetic instability [47], and tumorigenicity [48, 49], although these results have been challenged [48]. Multipotentiality, hence therapeutic potency, has been shown to diminish with serial passaging, with human bone-marrow derived MSCs progressively losing their potential for adipogenic and chondrogenic differentiation as the number of cell divisions increases [50]. Regardless of the protocol for culture expansion, MSCs undergo replicative senescence in culture, limiting their clinical applications [51]. In addition, expression of adhesion molecules and chemokines, and the ability to respond to chemokines, decline with time in culture [51].

Finally, almost immediate availability of autologous presumptive MSCs would allow immediate treatment of emergency conditions without depending on weeks of in vitro culture to collect equivalent therapeutic cells.

## The future: activating stem cells in their native environment?

Possibly, yes. The widespread presence of MSCs throughout vascularized organs equates to a reservoir of potentially therapeutic regenerative units throughout the body. This raises the possibility of using therapeutic strategies to ‘recruit’ or accelerate the regenerative capacity of resident MSCs following injury. The ‘activation’ of adult stem cells is known to be regulated, in part, by their neighboring cells [52–54]; manipulation of this communication through the local infiltration of growth factors or molecules integral to this interaction may represent such an approach, the ultimate goal being the manipulation and activation of the regenerative potential of both local stem cells and those recruited from distant sites. As MSCs are ubiquitous amongst vascular tissues, controlled activation of their potential would provide an alternative to purification and transplantation in cases where the native structural environment remains sufficiently intact to accommodate repair. In situ activation of MSCs could be harnessed to accelerate healing and facilitate an early return to function after a variety of musculoskeletal injuries. Other potential applications of activating resident MSCs could also include the treatment of osteoporosis and muscular dystrophies where local application of MSCs alone is not practical. In summary, the controlled activation of MSCs in their native environment will be an important future therapeutic approach.
